# Changes in cigarette smoking initiation, cessation, and relapse among U.S. adults: a comparison of two longitudinal samples

**DOI:** 10.1186/s12971-017-0121-3

**Published:** 2017-03-14

**Authors:** Zinan Yi, Maria E. Mayorga, Kristen Hassmiller Lich, Jennifer L. Pearson

**Affiliations:** 1 Operations Research Graduate Program, North Carolina State University, Raleigh, NC USA; 2 Department of Industrial and Systems Engineering, North Carolina State University, Raleigh, NC USA; 3 Gillings School of Global Public Health, University of North Carolina at Chapel Hill, 1105E McGavran-Greenberg HallCB #7411, Chapel Hill, NC 27599-7411 USA; 4 Schroeder Institute for Tobacco Research & Policy Studies, Truth Initiative, Washington, DC USA; 5 Department of Health, Behavior, and Society, Johns Hopkins Bloomberg School of Public Health, Baltimore, MD USA

**Keywords:** Smoking, Initiation, Cessation, Relapse, Cigarette

## Abstract

**Background:**

The tobacco epidemic in the U.S. has matured in the past decade. However, due to rapidly changing social policy and commercial environments, tailored prevention and interventions are needed to support further reduction in smoking.

**Methods:**

Using Tobacco Use Supplement to the Current Population Survey (TUS-CPS) 2002–2003 and 2010–2011 longitudinal cohorts, five smoking states are defined including daily-heavy, daily-light, non-daily, former and non-smoker. We quantified the changes between smoking states for the two longitudinal cohorts, and used a series of multivariable logistic regression models to examine the association of socio-demographic attributes and initial smoking states on smoking initiation, cessation, and relapse between waves within each cohort.

**Results:**

The prevalence of adult heavy smoking decreased from 9.9% (95% CI: 9.6%, 10.2%) in 2002 to 7.1% (95% CI: 6.9%, 7.4%) in 2010. Non-daily smokers were less likely to quit in the 2010–2011 cohort than the 2002–2003 cohort (37.0% vs. 44.9%). Gender, age group, smoker type, race and marital status exhibit similar patterns in terms of their association to the odds of initiation, cessation and relapse between the two cohorts, while education groups showed some inconsistent results between the two cohorts regarding the odds of cessation.

**Conclusions:**

Transitions between smoking states are complex and increasingly unstable, requiring a holistic, population-based perspective to understand the stocks and flows that ultimately dictate the public health impact of cigarette smoking behavior. This knowledge helps to identify groups in need of increased tobacco control prevention and intervention efforts.

## Background

Over the past 50 years, the cigarette smoking epidemic in the U.S. has declined thanks to changing tobacco control policy and smoking-related norms. [1] Over the last decade, youth and adult cigarette smoking has decreased significantly [1–3]. There has been a shift toward lighter smoking, with increasing prevalence of non-daily smoking [4, 5] and an overall decrease in cigarette consumption [6]. Despite these trends, smoking continues to be the leading cause of preventable disease and death in the U.S., accounting for more than 480,000 deaths every year [1]. Smoking, especially daily-heavy smoking, is concentrated among those with the least education and lowest incomes [7, 8]. As the nature of the U.S. smoking epidemic continues to change, it is important to understand dynamic smoking behaviors (e.g., initiation, cessation, and relapse) by sub-population to provide effective tobacco control interventions that decrease overall smoking prevalence while also decreasing smoking-related health disparities.

Reducing initiation, encouraging cessation, and reducing the high rate of relapse are continued challenges for tobacco control. While most first experiences with cigarette smoking occur in adolescence [1, 9], recent studies suggest that the average age of initiation in the U.S. has increased [8]. Studies and developmental theory suggest that establishment of regular, lifelong smoking patterns occurs not in adolescence, but after age 18, when individuals move away from family influences and gain legal access to tobacco products [10]. Race/ethnicity also plays a role in smoking initiation, with non-Hispanic blacks initiating later than non-Hispanic whites [11, 12]. Differences in quit attempts and cessation success are also evident by sociodemographic characteristics. Recently, the Centers for Disease Control and Prevention found that while more than half of adult smokers reported past-year quit attempts, only 6.2% of all smokers were not smoking 1 year later [13], with the majority of relapse occurring within the first week after quitting [14]. Young adults are more likely than older adults to try to quit, and to quit smoking successfully [1, 15]. While non-Hispanic black smokers make more attempts to quit, non-Hispanic white smokers are more successful at staying quit [16, 17]. To fully understand the smoking behavior dynamics which may lead to health disparities in the U.S. adult population, examining trends holistically by sociodemographic characteristics in initiation, cessation, and relapse is essential.

Most previous longitudinal studies of individuals’ changes in smoking status and intensity have focused on transitions in specific populations (e.g., current and former smokers [18], non-daily smokers [19], adolescent smokers [20–23], or individuals with major depressive disorders [24] or alcohol or drug use disorders [25]), sometimes in community-based or statewide samples [18, 26, 27]. Few studies have assessed smoking transitions using prospective data, which decreases problems of recall or temporality common in cross sectional data [20–23, 26–31]. Even fewer studies have examined changes in smoking status in the entire US adult population using nationally representative data [27–29]. In an analysis from 1990, McWhorter et al. examined predictors of smoking cessation and relapse using the U.S. NHANES I dataset (1971–1975). The authors found that smokers who are older, white, smoke fewer cigarettes per day, have a higher household income, and reported a hospitalization in the followup period were independent predictors of quitting [28]. In a more recent analysis of Canadian adult smokers, Bondy et al. examined the correlates of transitions between daily, non-daily, and former smoking over three six-month follow-ups. The authors found that smokers who reduced their smoking between time points one and two were more likely to be quit at time point three than those who did not reduce at time point two. However, reducing smoking between time points 1 and 2 also carried a greater risk of relapse to daily smoking compared to those who quit without reducing [27]. Recently, Weinberger et al. [29] examined smoking stability among U.S. adults between 2001 and 2005, with a focus on transitions between daily and non-daily smoking using a U.S. nationally representative longitudinal dataset. They found that smoking statuses were very stable between 2001 and 2005, while certain groups (men, adults who are younger,unmarried, have less education and identify as Hispanic) need increased intervention and prevention effort. While these and other studies present essential data on population-level smoking dynamics, each has drawbacks that limit generalizability, including: 1) utilizing data gathered before the rise of e-cigarettes and other non-traditional tobacco products [27–30]; 2) excluding data on all possible smoking transitions (e.g., initiation, cessation, and relapse) [27, 28, 31]; 3) exclusion of heaviness of smoking from analyses.

The 2002–2003 and 2010–2011 longitudinal datasets from the Tobacco Use Supplement to the Current Population Survey (TUS-CPS) provide an opportunity to examine transitions in cigarette smoking, including changes in non-smoking, smoking (divided into non-daily, daily-heavy, and daily-light smoking), and former smoking, in two large, nationally representative cohorts of U.S. adults. The primary aim of this study was to examine changes in smoking transitions across an 8-year period, based on smoking assessments among two cohorts (2002–2003 and 2010–2011) of adults. Secondary aims of this study were: 1) to describe changes in cigarette smoking initiation, cessation, and relapse by sociodemographic characteristics across the 2002–2003 and 2010–2011 cohorts; and 2) to describe changes in cessation and relapse by cigarette smoking intensity across the 2002–2003 and 2010–2011 cohorts. Illuminating changes in smoking transitions and identifying groups that are at elevated risk of initiation or relapse will inform prevention and treatment efforts.

## Methods

### Dataset

This analysis employs two de-identified, publicly available longitudinal datasets from the Tobacco Use Supplement to the Current Population Survey (TUS-CPS) from 2002 to 2003 (*N* = 15,846) [32] and 2010–2011 (*N* = 28,153) [33]. Detailed information on survey methods is available elsewhere [33]. Briefly, the purpose of the TUS-CPS is to monitor tobacco use behavior, attitudes, and norms at national and lower levels. The data are nationally representative of the civilian, non-institutionalized U.S. population and is collected using telephone and in person interviews [34].

### Measures

#### Socio-demographics

We grouped participants into three age groups (18–29, 30–44 and 45+) to allow for comparisons in different adult developmental stages (young, middle, and older adulthood) and to be consistent with similar studies [29]. While a more narrow definition of young adulthood would have been preferable, our granular approach to defining smoking status. did not allow for further division by age. Educational attainment was aggregated from 18 categories into three categories: less than high school (any with no diploma), high school degree/GED (high school grad-diploma or GED), and higher than high school (any college and higher). Marriage status was aggregated into two categories: currently married (married-spouse present or absent), and currently unmarried. Four race groups (non-Hispanic white, non-Hispanic black, Hispanic, and Other) are coded using information reported in two survey items.

#### Smoking states

We created five mutually exclusive smoking states: daily-heavy, daily-light, non-daily, former, and non-smokers. Respondents who respond “No” to “Have you ever smoked more than 100 cigarettes in your entire life?” were considered non-smokers. The other four smoker types were restricted to respondents who respond “Yes” to “Have you ever smoked more than 100 cigarettes in your entire life?” (i.e., they had smoked at least 100 lifetime cigarettes. Daily-heavy and daily-light smokers reported “everyday” to “Do you now smoke cigarettes every day, some days, or not at all?” with daily heavy smokers reporting consuming ≤ 10 cigarettes (consistent with prior definitions of light smoking [3, 4]), and daily-heavy smokers consuming >10 cigarettes per day to the question “On the average, about how many cigarettes do you now smoke each day?”. Non-daily smokers reported “Some days” to “Do you now smoke cigarettes every day, some days, or not at all?”, and were grouped regardless of the number of cigarettes they consumed on the days they smoked. Former smokers reported “Not at all” to “Do you now smoke cigarettes every day, some days, or not at all?”

#### Smoking transitions

We examined changes in these smoking states with a focus on initiation, cessation and relapse, defined as moving between the following smoking states between waves 1 and 2: “Initiation”: from non-smoking to any current smoking; “Cessation”: from any current to former smoking; “Relapse”: from former to any current smoking.

### Inclusion & exclusion criteria

To obtain our final samples of *N* = 15,410 (2002–2003) and *N* = 18,393 (2010–2011), we used the following exclusion criteria. First, we excluded *N* = 358 participants from the 2002–2003 dataset who were <18 years old in 2002 to confine our analyses to adults, as the 2010–2011 TUS-CPS did not enroll individuals <18. Second, we excluded participants (*N* = 78 in 2002–2003, *N* = 106 in 2010–2011) because their smoking state was indeterminate. Smoking state was “indeterminate” if a participant answered “refused” or “don’t know” to any of the following questions: “Have you ever smoked more than 100 cigarettes in your entire life?” or “Do you now smoke cigarettes every day, some days, or not at all?” in either wave. In total, 2.8% of the 2002–2003 and 0.6% of the 2010–2011 original samples were excluded due to these procedures. *N* = 9,654 observations were excluded from proxy respondents in the 2010–2011 dataset because the 2002–2003 dataset did not collect information by proxy, and full information about the target participant (e.g. number of cigarettes smoked per day) was not collected via proxy. Proxy responses were only collected in certain special situations (e.g. respondent is getting irritated) and proxies can only be taken for a subset of questions (for further details see report from Office of Disease Prevention and Health promotion [35]).

Some participants also reported inconsistent smoking state changes between survey waves. “Inconsistent” responses were defined as individuals who responded “yes” to “Have you ever smoked more than 100 cigarettes in your entire life?” in wave 1 but reported “no” to the same question in wave 2 (*N* = 1,020 in 2002–2003, *N* = 1,041 in 2010–2011). In other words, an “inconsistent” response came from an individual who reported to have smoked more than 100 lifetime cigarettes and went on to answer questions about frequency and quantity of smoking in wave 1, but reported never smoking more than 100 cigarettes in the following wave, a change which is infeasible. Among “inconsistent” responders, 70–75% self-reported as former smokers (had smoked 100 lifetime cigarettes but were currently not smoking) at wave 1, with the remaining 25–30% self-reporting as either non-daily or daily-light smokers at wave 1. Because most of these participants had identified as former smokers in 2002 or 2010, we assumed that their wave 1 response was correct and changed all their wave 2 smoking state from “non-smoker” (less than 100 lifetime cigarettes) to “former,” as they had all reported smoking at least 100 lifetime cigarettes in wave 1. While it is possible that some particpants could have misreported smoking at least 100 cigarettes in their lifetimes at wave 1, leading them to a series of inapplicable questions about smoking patterns, it is more likely that the majority of inconsistent responses at wave 2 came from individuals who noted the additional questions on smoking status in at wave 1 and preferred to reduce their responde burden at wave 2. In sensitivity analyses (analyses not shown), we compared our reclassification approach with results from an analysis where we excluded inconsistent responders and found no qualitative difference in our results.

### Statistical analysis

To account for the complex survey design and response rate, we used the main and replicate weights provided with the data as recommended for the TUS-CPS overlap samples in all analyses [33], with replicate weights derived using balanced repeated replication [36].

We estimated the proportion of participants that transitioned between smoking states from wave 1 to wave 2 and corresponding 95% confidence intervals (CI) around each estimate using PROC SURVEYFREQ in SAS (Version 9.3, SAS Institute, Cary, NC).

A series of multivariable logistic regression models were estimated to examine the association of socio-demographic attributes and initial smoking states with smoking transitions between waves for both cohorts using the PROC SURVEYLOGISTIC procedure in SAS. Each model included the primary independent variables of gender, age group, race/ethnicity, marital status and education. Results are presented as adjusted odds ratios (aOR) with corresponding 95% CIs. In addition, for any given measure of interest we test differences in proportions between the two cohorts, with the null hypothesis being that there is no difference between the two cohorts, then an association is statistically significant if the corresponding *p*-value is less than 0.05.

## Results

Table 1 presents weighted descriptive sociodemographic and smoking statistics for the analytic samples. Overall, the distribution of sex, age, and race/ethnicity were similar between the two cohorts. We observed a few differences in marital status, education and smoking state. In 2010 compared to 2002, a larger proportion of individuals were currently married and had completed at least some college, and a smaller proportion had not completed a high school degree. There were more non-smokers and fewer former and daily-heavy smokers in 2010 than in 2002. More specifically, the prevalence of adult heavy smoking decreased from 9.9% (95% CI: 9.6%, 10.2%) in 2002 to 7.1% (95% CI: 6.9%, 7.4%) in 2010.

**Table 1:** Socio-demographic characteristics and cigarette smoking state among participants in TUS-CPS 2002-2003 & 2010-2011

Time-invariant sociodemographic characteristics	2003 (%, 95%CI) (*n* = 15,410)		2010 (%, 95%CI) (*n* = 18,499)	
Sex				
Male	48.2 (48.1, 48.3)		48.3 (48.3, 48.3)	
Female	51.8 (51.7, 51.9)		51.7 (51.7, 51.7)	
Age				
18-29	21.1 (20.9, 21.4)		21.1 (20.9, 21.3)	
30-44	31.2 (31.1, 31.4)		27.2 (27.0, 27.4)	
45+	47.6 (47.5, 47.7)		51.8 (51.6, 51.7)	
Marital Status				
Currently married	44.9 (44.4, 45.4)		48.3 (47.9, 48.8)	
Currently not married	55.1 (54.6, 55.6)		51.7 (51.2, 52.1)	
Race/Ethnicity				
Non-Hispanic White	70.5 (70.4, 70.7)		68.0 (68.0, 68.1)	
Non-Hispanic Black	11.4 (11.3, 11.5)		11.4 (11.4, 11.4)	
Hispanic	12.3 (12.2, 12.5)		13.9 (13.9, 14.0)	
Asian/other	5.7 (5.7, 5.8)		6.6 (6.6, 6.6)	
Time-varying sociodemographic characteristics	2002 (%, 95%CI)	2003 (%, 95%CI)	2010 (%, 95%CI)	2011 (%, 95%CI)
Education				
Less than high school degree	18.6 (18.2, 18.9)	16.6 (16.2, 17.1)	13.1 (12.8, 13.4)	11.7 (11.3, 12.0)
High school degree/GED	29.3 (28.8, 29.8)	29.6 (29.0, 30.1)	28.7 (28.2, 29.0)	28.0 (27.4, 28.3)
> high school degree^a^	52.1 (51.5, 52.7)	53.8 (53.1, 54.4)	58.2 (57.8, 58.8)	60.4 (60.0, 60.9)
18-year-old smoking prevalence				
Non-smoker	87.8 (83.1, 92.1)		92.5 (89.6, 95.3)	
Smoking status				
Daily heavy smoker	9.9 (9.6, 10.2)	9.2 (8.9, 9.5)	7.1 (6.9, 7.4)	6.6 (6.3, 6.8)
Daily light smoker	4.9 (4.6, 5.2)	4.9 (4.6, 5.2)	5.1 (4.9, 5.3)	5.0 (4.7, 5.2)
Non-daily smoker	4.2 (4.0, 4.5)	3.4 (3.2, 3.6)	3.4 (3.2, 3.6)	2.7 (2.6, 2.9)
Former smoker	22.2 (21.8, 22.7)	27.2 (26.7, 27.7)	19.5 (19.3, 19.9)	25.8 (25.4, 26.2)
Non-smoker	58.7 (58.1, 59.4)	55.3 (54.6, 55.9)	64.6 (64.3, 65.3)	60.0 (59.5, 60.5)

^a^Includes some college/associate's degree/some other degree, and bachelor's degree or higher, *CI* Confidence Interval

Table 2 presents the prevalence of smoking states and transitions in smoking states between waves for each cohort overall and by age and race group. For the three age groups in both cohorts, as age increases, the proportion of former smokers increases while the proportions of daily-light, non-daily, and non-smokers decrease. The age group with the highest proportion of daily-heavy smokers in 2002–2003 was the 30–44 year olds. This is the same birth cohort (now 45+) who also had the highest proportion of daily-heavy smokers in 2010–2011. Also, the older age groups had lower percentages of initiation, cessation and relapse than younger age groups in both cohorts.

**Table 2:** Prevalence and changes in smoking status between waves, by age and race; TUS-CPS 2002-2003&2010-2011

	2002-2003 (%, 95% CI)						2010 -2011 (%, 95% CI)					
	Age 18-29	30-44	45+	NHW	NHB	Hispanic	Age 18-29	30-44	45+	NHW	NHB	Hispanic
Smoking Status												
Daily heavy smoker	7.8 (7.0, 8.6)	9.9 (9.4, 10.4)	9.3 (9.0, 9.6)	11.3 (10.9, 11.6)	5.0 (4.2, 5.7)	2.7 (2.1, 3.4)	5.7 (5.0, 6.4)	6.9 (6.5, 7.3)	7.9 (7.6, 8.1)	9.0 (8.7, 9.3)	4.0 (3.5, 4.5)	2.3 (1.9, 2.8)
Daily light smoker	7.6 (6.8, 8.5)	4.7 (4.4, 5.1)	3.8 (3.6, 4.0)	4.6 (4.2, 4.9)	7.8 (6.9, 8.7)	4.7 (3.8, 5.6)	6.1 (5.4, 6.8)	5.6 (5.2, 6.0)	4.4 (4.2, 4.7)	4.9 (4.7, 5.2)	6.5 (5.9, 7.2)	4.5 (3.9, 5.0)
Non-daily smoker	4.4 (3.8, 5.0)	3.8 (3.5, 4.2)	2.7 (2.5, 2.9)	3.0 (2.8, 3.2)	4.6 (3.8, 5.4)	5.0 (4.0, 5.9)	5.1 (4.5, 5.7)	4.0 (3.6, 4.3)	2.4 (2.2, 2.6)	3.0 (2.8, 3.2)	4.4 (3.8, 5.1)	4.9 (4.3, 5.6)
Former smoker	15.0 (13.9, 16.1)	22.2 (21.4, 23.0)	35.9 (35.3, 36.5)	30.4 (29.8, 30.9)	21.4 (19.9, 22.9)	18.0 (16.6, 19.4)	7.7 (7.1, 8.3)	14.7 (14.1, 15.2)	27.0 (26.5, 27.4)	23.3 (23.0, 23.7)	11.7 (10.8, 12.5)	11.0 (10.2, 11.8)
Non smoker	65.3 (63.7, 66.8)	59.3 (58.5, 60.1)	48.2 (47.6, 48.9)	50.8 (50.2, 51.4)	61.2 (59.7, 62.8)	69.6 (67.8, 71.4)	75.3 (74.2, 76.5)	68.9 (68.1, 69.7)	58.3 (57.9, 58.8)	59.8 (59.2, 60.3)	73.4 (72.1, 74.6)	77.2 (76.0, 78.4)
Changes of Smoking Status												
% Cessation	26.9 (23.7, 30.1)	24.0 (22.2, 25.8)	18.6 (17.2, 20.0)	18.5 (17.6, 19.5)	30.3 (25.7, 34.9)	37.3 (31.7, 42.9)	30.4 (27.1, 33.6)	27.2 (25.4, 29.1)	19.3 (18.1, 20.5)	21.6 (20.4, 22.7)	26.5 (23.4, 76.6)	35.3 (31.5, 39.0)
% Relapse	19.6 (15.9, 23.2)	6.7 (5.6, 7.7)	4.6 (4.1, 5.0)	6.5 (5.9, 7.1)	6.8 (4.2, 9.3)	5.9 (3.4, 8.3)	15.0 (11.8, 18.2)	7.6 (6.4, 8.7)	3.6 (3.2, 4.0)	5.0 (4.4, 5.5)	6.9 (4.9, 9.0)	9.0 (6.9, 11.1)
% Initiation	3.8 (3.0, 4.6)	2.1 (1.7, 2.5)	1.3 (1.2, 1.5)	1.9 (1.7, 2.2)	3.4 (2.5, 4.3)	3.1 (2.1, 4.1)	3.2 (2.7, 3.8)	2.1 (1.8, 2.3)	1.4 (1.3, 1.6)	2.0 (1.8, 2.2)	2.9 (2.4, 3.4)	2.3 (1.7, 2.8)

*NHW* non-Hispanic whites, *NHB* non-Hispanic blacks, *Hisp* Hispanic, *CI* Confidence Interval

Comparing transition trends by race/ethnicity, non-Hispanic whites had the highest proportion of daily-heavy and former smokers. Non-Hispanic blacks had the highest proportion of daily-light smokers, and Hispanics had the highest proportion of non-daily and non-smokers. Regarding changes in smoking state, there is evidence of low but non-trivial initiation in all race/ethnicity groups, with initiation highest among non-Hispanic blacks and cessation highest among Hispanics in both cohorts. There is no consistent pattern of of relapse by race/ethnicity change across cohorts.

**Fig. 1:**
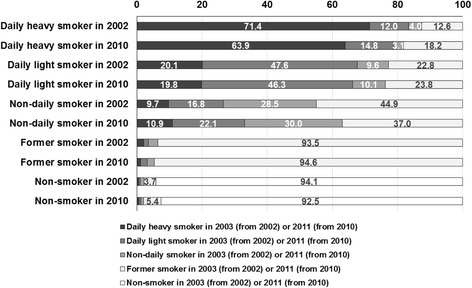
Proportion of individuals transitioning from a smoking status in 2002 (2010) to 2003 (2011)

Figure 1 illustrates proportion of individual who transition from a smoking status in wave 1 to a smoking status in wave 2 in the 2002–2003 and 2010–2011 cohorts (percentages <3% not shown numerically). The figure is read left to right. For example top bar shows that of those that were daily smokers in 2002, 71.4% remained heavy smokers in 2003. We observed no statistically significant change (*p* > .05) in initiation patterns between the cohorts at the overall population level, with 2.2% and 2.1% of non-smokers in wave 1 initiating smoking in wave 2 in 2002–2003 and in 2010–2011, respectively, though some non-smokers initiated and quit (thereby becoming former smokers) within the year (3.7% and 5.4% in 2002–2003 versus 2010–2011). The largest changes in cessation transtions were observed among non-daily smokers: 37.0% (95% CI: 34.4%–39.5%) of non-daily smokers stopped smoking in 2010–2011, while 44.9% (95% CI: 42.1%–47.8%) of non-daily smokers stopped smoking in 2002–2003. In contrast, for daily-heavy smokers, the proportion quitting in 2010–2011 was higher than in 2002–2003; 18.2% (95% CI: 16.7%–19.8%) and 12.6% (95% CI: 11.5%–13.6%), respectively. For daily-light smokers, the difference in cessation between the two cohorts was not statistically significant. Finally, no significant differences were observed in terms of relapse among former smokers between the two cohorts.

By gender, age, and marital status, the odds of smoking initiation remained similar between the two cohorts, with men, younger adults (compared to adults over age 45), and unmarried adults having higher odds of cigarette smoking initiation (Table 3). However, compared to 2002–2003, the relationship in 2010–2011 between race/ethnicity, education, and smoking initiation changed. In 2002–2003, non-Hispanic blacks had greater odds of smoking initiation than non-Hispanic whites (aOR: 1.42, 95% CI: 1.04–1.93); in 2010–2011, there was no statistically significant difference. Additionally, relative to non-Hispanic whites, the odds of initiation for Hispanics decreased between 2002–2003 (aOR: 1.19, 95% CI: 0.81–1.74) and 2010–2011 (aOR: 0.69, 95% CI: 0.50–0.96). By education, we observed a widening of the effect of education on smoking initiation. In 2002–2003, adults with less than a high school education had 1.5 (95% CI:1.17,2.13) times greater odds of initiation than adults with more than a high school education, and there was no difference in initiation comparing adults with a high school degree/GED to those with more than a high school degree. In 2010–2011, adults with the least education had nearly 3.4 times the odds (aOR: 3.39, 95% CI: 2.52–4.56) of initiating smoking compared to those with the most education. Additionally, the association between initiation and having a high school degree/GED was significant (aOR: 2.85, 95% CI:2.34–3.48).

**Table 3:** Multivariable logistic regression of the association of smoker’s characteristics to initiation, cessation and relapse; TUS-CPS:2002-2003&2010-2011

	Odds of initiation (OR, (95% CI))		2002-2003		2010-2011		Odds of cessation (OR, (95% CI))		2002-2003		2010-2011		Odds of relapse (OR, (95% CI))		2002-2003		2010-2011	
Characteristics	2002-2003 (*N* = 146)	2010-2011 (*N* = 203)	*χ* ^2^	p-value	*χ* ^2^	p-value	2002-2003 (*N* = 596)	2010-2011 (*N* = 625)	*χ* ^2^	p-value	*χ* ^2^	p-value	2002-2003 (*N* = 220)	2010-2011 (*N* = 184)	*χ* ^2^	p-value	*χ* ^2^	p-value
Gender			46.3	0.0002	46.33	<0.0001			0.95	0.3289	0.0438	0.8343			2.52	0.1123	0.06	0.8097
Male	1	1					1	1					1	1				
Female	0.66(0.53, 0.84)*	0.53(0.44, 0.64)*					1.07 (0.93, 1.23)	1.01 (0.91, 1.12)					1.14 (0.96, 1.35)	0.98 (0.82, 1.16)				
Age			40.6	<0.0001	40.59	<0.0001			18.86	<0.0001	45.12	<0.0001			257.17	<0.0001	150.11	<0.0001
18-29	2.11(1.66, 2.67)*	1.90(1.53, 2.35)*					1.38 (1.17, 1.63)*	1.71 (1.42, 2.06)*					5.66 (4.49, 7.14)*	4.42 (3.36, 5.82)*				
30-44	1.61(1.29, 2.02)*	1.66(1.35, 2.04)*					1.26 (1.09, 1.46)*	1.38 (1.22, 1.56)*					1.65 (1.34, 2.03)*	2.47 (2.01, 3.03)*				
45+	1	1					1	1					1	1				
Race			27.6	0.0001	29.5	<0.0001			26.59	<0.0001	39.3	<0.0001			2.88	0.41	11.83	0.008
NHW	1	1					1	1					1	1				
NHB	1.42(1.04, 1.93)*	1.05(0.84, 1.33)					1.49 (1.18, 1.87)*	1.41 (1.18, 1.69)*					1.07 (0.66, 1.74)	1.33 (0.94, 1.87)				
Hispanic	1.19(0.81, 1.74)	0.69(0.50, 0.96)*					1.65 (1.26, 2.15)*	1.62 (1.33, 1.98)*					0.67 (0.42, 1.07)	1.54 (1.16, 2.04)*				
Other	0.31(0.17, 0.56)*	0.31(0.19, 0.51)*					1.62 (1.20, 2.18)*	1.51 (1.21, 1.89)*					0.90 (0.56, 1.45)	0.70 (0.42, 1.19)				
Marital status			56.1	<0.0001	56.15	<0.0001			40.51	<0.0001	34.57	<0.0001			1.52	0.2176	69.95	<0.0001
Married	1	1					1	1					1	1				
Unmarried	1.66(1.28, 2.14)*	1.96(1.65, 2.34)*					0.68 (0.61, 0.77)*	0.71 (0.63, 0.79)*					0.86 (0.69, 1.09)	2.11 (1.77, 2.51)*				
Education			123.2	0.0002	126.4	<0.0001			31.88	<0.0001	50.69	<0.0001			27.42	<0.0001	6.15	0.0461
< High school	1.58(1.17, 2.13)*	3.39(2.52, 4.56)*					1.59 (1.35, 1.87)*	0.68 (0.56, 0.82)*					1.95 (1.43, 2.66)*	1.30 (1.03, 1.65)*				
High school degree/GED	1.09(0.85, 1.38)	2.85(2.34, 3.48)*					0.95 (0.84, 1.07)	0.62 (0.55, 0.71)*					1.78 (1.46, 2.17)*	1.17 (0.96, 1.43)				
> High school	1	1					1	1					1	1				
Smoker Type									444.44	<0.0001	101.38	<0.0001						
Daily heavy	-	-					1	1					-	-				
Daily light	-	-					1.79 (1.55, 2.07)*	1.21 (1.04, 1.40)*					-	-				
Non-daily	-	-					5.11 (4.39, 5.95)*	2.11 (1.81, 2.46)*					-	-				

*: *p* < 0.05. *OR* Odds Ratio. N is unweighted. The results are all weighted. *CI* Confidence Interval

The relationship between socio-demographics and smoking cessation were similar in the 2002–2003 and 2010–2011 cohorts (Table 3). Gender was not related to smoking cessation, age was inversely related to cessation, and unmarried adults had lower odds of cessation in both cohorts. By race/ethnicity, the odds of cessation were the highest among Hispanics compared to non-Hispanic whites in both cohorts. By smoking state, the odds of cessation were the highest among non-daily smokers and lowest among daily-heavy smokers. The only difference observed between the two cohorts was by education. In 2002–2003, adults with less than a high school degree had a greater odds of cessation (aOR: 1.59, 95% CI: 1.35–1.87) than those higher levels of education. There was no observed difference in cessation comparing adults with a high school degree/GED to those who had more than a high school degree. In 2010–2011, this relationship had flipped, with lower odds of cessation associated with having less than a high school degree (aOR: 0.68, 95% CI: 0.56–0.82) or a high school degree/GED (aOR: 0.62, 95% CI: 0.55–0.71) compared to those with the highest level of education.

Younger age was associated with an elevated odds of relapse to smoking in both cohorts. Lower education also had a similar association with relapse in both cohorts, though the strength of the association was attenuated in 2010–2011 cohort compared to 2002–2003. The association between marital status, race/ethnicity, and relapse were different in 2010–2011 and 2002–2003. The odds of relapse were 2.11 times higher (95% CI: 1.77–2.51) for unmarried than married adults in 2010–2011. While there was no relationship between race/ethnicity and relapse in 2002–2003, Hispanics had a higher odds of relapse compared to non-Hispanic whites (aOR: 1.54, 95% CI:1.16–2.04); non-Hispanic blacks also showed an increased but non-significant odds of relapse in 2010–2011. Gender was not associated with relapse in either cohort.

## Discussion

Transitions between smoking states are complex and increasingly unstable, requiring a holistic, population-based perspective to understand the stocks and flows that ultimately dictate the public health impact of cigarette smoking behavior. Consistent with cross sectional research, we found a decrease in overall smoking [1–3], a substantial decrease in daily-heavy smoking, and a shift toward daily-light and non-daily smoking. Studying transitions over time across the two cohorts provides further insights into these changes. First, daily-heavy smoking was less stable in 2010, with greater movement to daily-light and former smoking than among daily-heavy smokers in 2002. This is consistent with existing evidence and prior research showing that cigarette consumption has decreased [6]. While we observed reduction in daily-heavy smokers, we also observed escalation among non-daily smokers across the cohorts, with increases in transition from non-daily to all other current smoking states. These changes could be due to several population-level approaches to tobacco control and changes in product availability. First, while most quit attempts are unaided [37], the increased availability of FDA-approved smoking cessation treatments and the widespread use of e-cigarettes to quit or cut down could have encouraged reduction or cessation among daily-heavy smokers in 2010 compared to 2002. Conversely, it is also possible that e-cigarette use could have increased transition to daily smoking among non-daily smokers in 2010. Second, it is possible that the expansion of smoke-free indoor air laws, heightened anti-smoking norms, and increased cigarette taxes have made smoking more difficult and expensive. Indeed, researchers have observed that smokers have learned to extract more nicotine from cigarettes as their opportunities to smoke have decreased, allowing individuals to maintain similar levels of nicotine dependence though they have decreased their daily cigarette consumption [38]. Evidence from product evaluations also suggest that cigarette nicotine yields have increased over time, perhaps as a response to restricted smoking opportunities [39, 40]. These changes to tobacco control policy and product design may have increased the amount of time that individuals spend as a non-daily smokers before they transition to cessation or escalate to daily smoking. Policy and behavioral interventions to encourage non-daily smokers to quit may have significant impact on overall adult smoking prevalence in the U.S. Future research should examine drivers of reduction and cessation among daily-heavy smokers and escalation among non-daily smokers.

Overall, sociodemographic trends in smoking initiation, cessation, and relapse showed consistencies between the two cohorts. Gender, age group, and smoking intensities exhibited the same pattern of association to the odds of initiation, cessation, and relapse between the two cohorts. For example, gender played a significant role in initiation, but not in cessation or relapse, with females being less likely to initiate than males. Younger age groups were more likely to transition between states than older groups [41]. Specifically, age 19–29 had a very high odds of relapse to smoking, which may be related to young adults’ social smoking behavior causing difficulty in cessation and in staying abstinent [42]. The impact of marital status on smoking behavior was relatively unchanged between the two cohorts. Married adults displayed "better" smoking behavior, having lower odds of initiation and relapse, and higher odds of cessation, implying that marriage may have a positive impact on smoking behavior [43–45]. Education groups showed some inconsistent results between the two cohorts regarding the odds of cessation. In both cohorts, individuals with lower education levels had a higher odds of initiation and relapse than individuals with higher education levels, possibly reflecting differences in understanding of the hazards of smoking, exposure to smoking-related health information, coverage of smoke-free indoor air laws, and access to smoking prevention interventions and cessation aids [46].

### Limitations

While complex tobacco use profiles that include products such as cigars, e-cigarettes, and hookah are likely more reflective of individuals’ tobacco use behaviors, the TUS-CPS 2002–2003 only included detailed questions on cigarette smoking. While the TUS-CPS 2010–2011 collected more information on the use of new products such as e-cigarettes, we were not able to compare differences in other tobacco products use between the two cohorts. Furthermore, smoking transitions were based on follow-up over 12 months; thus, the potential for relapse beyond 12 months, or for cessation and relapse within 12 months is not captured in our analysis. Lastly, while occupation/employment status could have been a useful addition, we did not include it for several reasons: first, the addition of this variable did not improve model fit while reducing parsimony; second, 22% of participants were missing self-reported occupation/employment responses; and third, to be consistent with similar analyses [29].

## Conclusions

Transitions between smoking states are complex and increasingly unstable, requiring a holistic, population-based perspective to understand the stocks and flows that ultimately dictate the public health impact of cigarette smoking behavior. Our analysis can inform targeted interventions, suggesting that initiation efforts should target young adults, males [29, 47], those who are unmarried [48], non-Hispanic blacks, and individuals with lower education levels [29], as these groups are at higher risk of initiating smoking. Interventions encouraing cessation should target older and non-Hispanic white adults [29, 49], unmarried individuals, those with a high school degree/GED, and heavier smoking groups because these individuals are less likely to quit successfully. Prevention of relapse should focus on younger, married, and less educated groups that are most likely to relapse, consistent with other studies [29, 50, 51]. Further research should investigate the extent to which these transitions in smoking are offset or bolstered by other forms of tobacco use. While the direction of change is good, efforts to sustain changes made over the past decade should be bolstered.
